# Macrophage Metabolic Reprogramming in Rheumatoid Arthritis: Pathogenic Mechanisms and Therapeutic Implications

**DOI:** 10.3390/cells15131166

**Published:** 2026-06-26

**Authors:** Longping Chen, Siyuan Leng, Xin Liu, Junlan Zhang, Fang Zhao, Zeyu Hu, Xiong Cai, Ye Lin

**Affiliations:** 1 School of Chinese Medical Sciences, Hunan University of Chinese Medicine, Changsha 410208, China; chenxx@stu.hnucm.edu.cn; 2 School of Pharmacy, Hunan University of Chinese Medicine, Changsha 410208, China; liu.in@stu.hnucm.edu.cn; 3 Institute of Innovation and Applied Research in Chinese Medicine, Hunan University of Chinese Medicine, Changsha 410208, China; siyuan_leng@stu.hnucm.edu.cn (S.L.); 18711152094@163.com (J.Z.); zhaofanglucky9@163.com (F.Z.); huzy1234@stu.hnucm.edu.cn (Z.H.)

**Keywords:** rheumatoid arthritis, macrophages, metabolic reprogramming, immunometabolism, synovial microenvironment

## Abstract

Rheumatoid arthritis (RA) is a chronic autoimmune disease characterised by persistent synovitis, progressive cartilage destruction and bone erosion. Recent advances in single-cell and spatial omics, together with immunometabolic studies, have revealed marked state heterogeneity among synovial macrophages in RA. Their metabolic reprogramming appears to sustain pathogenic cellular states, drive aberrant intercellular communication and impair the resolution of inflammation. Rather than acting as an independent initiating factor, it more likely operates as a downstream amplifier of disease. In this review, we outline the principal functional states and metabolic features of synovial macrophages in health and RA. We focus on how the rewiring of glucose, lipid and amino acid metabolism links inflammatory transcription, tissue remodelling and bone destruction. These connections are mediated by metabolic enzymes, metabolic intermediates, redox regulation and epigenetic modifications. We further summarise the immunometabolic effects of currently available antirheumatic drugs. We also appraise the preclinical evidence and translational limitations of metabolic pathway inhibitors, natural products and nanodelivery systems. It should be noted that most existing evidence still relies on in vitro polarisation systems and rodent models. Validation of metabolic flux, cell-state specificity and causal relationships in human synovium remains limited. As a narrative review focused on recent studies of synovial macrophage metabolism in health and inflammation, this work aims to delineate how metabolic reprogramming shapes the phenotypic heterogeneity and pathogenic functions of macrophages in RA. It also seeks to appraise the potential value and current boundaries of evidence for therapeutically targeting macrophage metabolism.

## 1. Introduction

Rheumatoid arthritis (RA) is a chronic, systemic autoimmune disease characterized by persistent synovial inflammation, synovial hyperplasia, and progressive destruction of cartilage and subchondral bone. Clinically, RA presents with joint pain, swelling, and deformity that profoundly impair quality of life and work capacity [1,2]. Epidemiological studies indicate that RA affects approximately 0.5–1% of the global population, with a 2- to 3-fold higher prevalence in women than in men and incidence rising with age [3,4]. Although biologic and targeted synthetic disease-modifying antirheumatic drugs (b/tsDMARDs) have substantially improved outcomes, a substantial proportion of patients fail to achieve sustained remission or progress to difficult-to-treat RA (D2T RA), indicating that key pathogenic mechanisms remain to be elucidated [5,6]. Current therapies largely target inflammatory mediators and immune signalling pathways. Macrophage metabolic reprogramming, however, is increasingly viewed as more than a passive consequence of inflammation. It may stabilize pathogenic macrophage states and perpetuate chronic synovitis, yet remains largely unaddressed by existing treatments.

The emergence of immunometabolism has provided a new conceptual framework for reinterpreting RA pathogenesis [7]. Growing evidence indicates that metabolism is not merely a passive process that supplies energy to immune cells, but actively shapes signal transduction, epigenetic modifications, and cell fate decisions through metabolites, enzyme activities, and shifts in metabolic flux [8,9,10,11]. In RA, immune-cell metabolic reprogramming has been implicated in sustaining inflammatory states and tissue-destructive interactions, with synovial macrophages serving as a central link between metabolic stress and pathological immune activation.

Macrophages are broadly distributed throughout the body and constitute a core component of the innate immune system, with essential roles in the clearance of pathogens and cellular debris, antigen presentation, regulation of inflammatory responses, tissue repair, and maintenance of homeostasis [12,13,14]. Compared with other immune cells, macrophages display remarkable heterogeneity and functional plasticity, enabling rapid adaptive responses to local microenvironmental cues, including hypoxia, nutrient limitation, acidification, and pro-inflammatory cytokines. Within the RA synovium, this adaptation is not merely a survival strategy but is coupled with profound metabolic reprogramming encompassing enhanced glucose metabolism, mitochondrial dysfunction, dysregulated lipid metabolism, and remodeled amino acid metabolism [15]. These metabolic alterations not only meet the energetic and biosynthetic demands of macrophages but also fundamentally shape their polarization, inflammatory phenotype, and pathological crosstalk with fibroblast-like synoviocytes (FLS), T cells, and osteoclasts, thereby driving the amplification of synovial inflammation, pannus formation, and bone erosion.

Thus, macrophage metabolic reprogramming is not merely a consequence of RA-associated inflammation but also contributes to sustaining pathogenic macrophage states and persistent synovitis. Targeting this process has therefore attracted increasing interest as a potential therapeutic approach in RA. However, a comprehensive synthesis of its molecular contribution to RA progression remains lacking. To address this gap, we focus on the functional states and metabolic adaptations of macrophages in RA and examine how alterations in glucose, lipid and amino acid metabolism contribute to disease progression within the synovial microenvironment. We also discuss emerging strategies for modulating macrophage metabolic reprogramming and critically consider their translational potential. Our goal is to provide a mechanistic framework for understanding RA pathogenesis and to facilitate the identification of potential therapeutic targets.

## 2. Methodology

Relevant literature was identified through comprehensive searches of PubMed, Web of Science, and Google Scholar using combinations of keywords related to rheumatoid arthritis, macrophage polarization, immunometabolism, glycolysis, lipid metabolism, amino acid metabolism, natural products, and nanomedicine. The search primarily focused on articles published between April 2021 and April 2026. Articles published in English and directly relevant to the scope of this review were included. Studies were selected based on scientific quality, novelty, and their relevance to the discussed molecular mechanisms and therapeutic implications. Priority was given to recent publications, particularly those utilizing single-cell RNA sequencing and spatial transcriptomics to investigate macrophage immunometabolism within the RA synovium, alongside highly cited foundational studies in the field.

## 3. Macrophage Phenotypes and Functions

### 3.1. Macrophage Phenotypes Associated with RA

RA is a chronic systemic autoimmune disease. It is characterised by persistent synovial inflammation, immune cell infiltration and progressive destruction of joint architecture [4,16]. Within this pathological process, macrophages serve as central effector cells of the innate immune system. They play a pivotal role in initiating inflammatory cascades and driving joint and bone destruction [17,18,19,20]. Synovial tissue macrophages (STMs) in RA exhibit pronounced heterogeneity and plasticity. Their functional states are jointly shaped by inflammatory cytokines, immune complexes, hypoxia and the local metabolic milieu. Heterogeneity is reflected in differences in phenotype, transcriptional profile and function. These differences vary across tissue origins, anatomical locations and disease stages of RA. Plasticity, in turn, refers to the capacity of macrophages to transition dynamically between activation states. Such transitions occur in response to changes in the local microenvironment, allowing macrophages to adapt to varying immunological and tissue demands.

Macrophage activation states have long been broadly categorised into two subtypes. These are classically activated “M1 macrophages” (pro-inflammatory) and alternatively activated “M2 macrophages” (anti-inflammatory) [21]. However, this view has been challenged by recent advances in single-cell transcriptomics, spatial omics and lineage-tracing technologies. Accumulating evidence indicates that RA synovial macrophages do not segregate into two discrete M1 or M2 states. Instead, they more closely resemble a continuous functional spectrum [22]. Consequently, the M1/M2 binary classification retains conceptual heuristic value. Yet it can no longer fully capture the true biological states of macrophages within the inflamed RA synovium.

Although the traditional M1/M2 framework cannot fully reflect the in vivo heterogeneity of human RA synovial macrophages, it remains useful in simplified settings. These include animal studies and in vitro pharmacological investigations, where it helps summarise the principal functional tendencies of macrophages. In RA-related preclinical research, the M1-like phenotype is typically associated with pro-inflammatory cytokine production, antigen presentation, and amplification of synovitis. It also drives activation of T cells and FLS. By contrast, the M2a, M2b and M2c-like phenotypes reflect varying degrees of immunoregulation, apoptotic cell clearance, tissue repair and inflammation resolution. Within chronic inflammatory environments, mixed states may also emerge that co-express both pro-inflammatory and reparative markers. Beyond these classical polarisation phenotypes, arthritis-associated osteoclastogenic macrophages (AtoMs) represent a functional subset with strong relevance to RA. They can serve as osteoclast precursors and participate in inflammation and bone erosion. The inducing factors, markers and functions of these representative phenotypes are summarised in Table 1.

It should be emphasised that these polarisation phenotypes are simplified descriptions. They are primarily derived from in vitro stimulation or animal models. As such, they cannot be directly equated with the true macrophage subsets present in human RA synovium. Single-cell transcriptomic and spatial studies have further identified distinct STM states. These states are associated with active inflammation, bone destruction and sustained remission. Accordingly, when discussing experimental models, this review uses “M1-like” or “M2-like” to describe marker-defined functional tendencies. In the context of human RA synovium, preference is given instead to the STM subset designations defined by single-cell or spatial studies.

### 3.2. Heterogeneity and Function of Synovial Macrophages in RA

Synovial macrophages are not merely downstream effectors of the RA inflammatory response. Rather, they act as dynamic regulators that link the breakdown of synovial homeostasis, the sustained recruitment of immune cells, the amplification of inflammation and the progression of bone destruction. Anatomically, the synovium is broadly divided into a lining layer and a sublining layer. Under healthy physiological conditions, the lining layer is composed predominantly of tissue-resident macrophages that form a dual physical and immunological barrier. These cells adopt anti-inflammatory and tissue-protective phenotypes, rely chiefly on oxidative phosphorylation, and carry out functions such as debris clearance, inflammation suppression and the maintenance of tissue homeostasis [36]. With the onset of RA, this homeostatic niche is disrupted by hypoxia, heightened oxidative stress, persistently elevated pro-inflammatory cytokines and autoantibody-related immune activation. As a consequence, the resident macrophages that once preserved homeostasis undergo phenotypic and functional reprogramming. Locally, they release abundant chemokines (such as IL-8 and CCL2) alongside danger-associated molecular patterns (such as S100A9). These mediators potently recruit peripheral blood monocytes, neutrophils and other inflammatory cells, which then transmigrate across the endothelium into the sublining layer [17]. Preclinical and early-RA studies suggest that loss of the homeostatic programme in synovial macrophages may precede the onset of clinical symptoms; whether this constitutes an independent disease-initiating factor, however, remains unclear [18,19].

Single-cell transcriptomic and high-dimensional immunophenotyping analyses further demonstrate that human STMs cannot be adequately captured by the traditional M1/M2 dichotomy. Instead, they occupy a continuum of functional states linked to disease activity, bone erosion, the risk of flare after treatment tapering or withdrawal, and sustained remission [19,37,38,39]. On the basis of MER tyrosine kinase (MerTK) and mannose receptor-1 (CD206) expression, human STMs can be broadly grouped into MerTK^+^CD206^+^ and MerTK^−^CD206^−^ populations [17]. MerTK^+^CD206^+^ STMs are more abundant in healthy synovium and in RA synovium during sustained remission, and they encompass tissue-resident subsets such as TREM2^+^CX3CR1^+^FOLR2^+^ and LYVE1^+^FOLR2^+^ cells [39,40]. These macrophages are chiefly engaged in clearing apoptotic cells and secreting tissue-reparative mediators (such as Resolvin D1 and IL-10). They also prompt FLS to secrete TGF-β1, thereby promoting inflammation resolution and the restoration of tissue homeostasis [15,41,42]. Recent spatial imaging studies reveal that the perivascular LYVE1^+^CD206^+^ resident macrophage network in the sublining is disrupted in active RA. In csDMARD good responders, by contrast, this network is partially restored with treatment, accompanied by the re-establishment of homeostatic crosstalk with fibroblasts and vascular cells. Such findings suggest that LYVE1^+^ macrophages may contribute to restoration of the synovial niche following treatment response [37]. In addition, the transition from high to low TREM2 expression, possibly driven by anti-citrullinated protein antibodies (ACPAs), not only marks the collapse of the immune barrier in the lining layer but also represents a key early node in the initiation of synovitis. The molecular mechanisms underlying this direct conversion, however, remain to be clarified [17,43,44].

**Figure 1:**
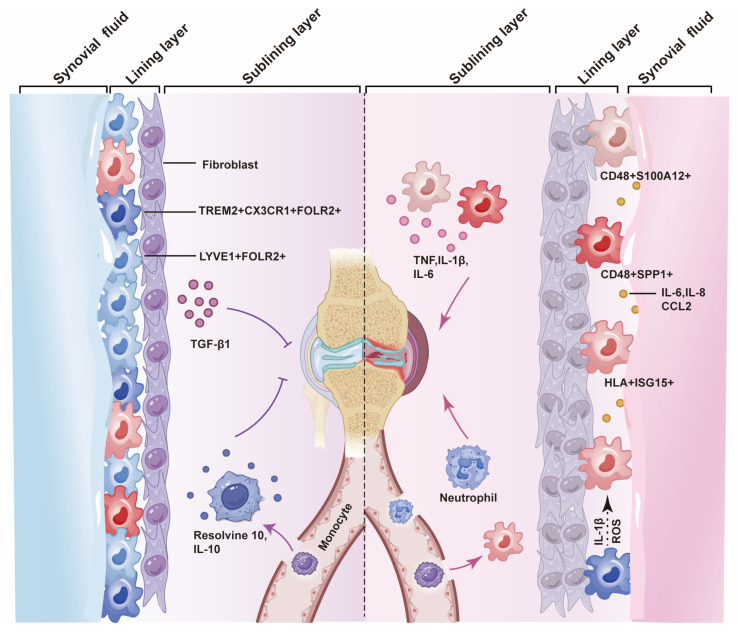
Heterogeneity and functional evolution of synovial macrophages in RA. Under physiological conditions, the synovial lining layer is enriched with tissue resident macrophages expressing high levels of TREM2 and MerTK, which preserve joint immune homeostasis and barrier integrity through efferocytosis of apoptotic cells and secretion of anti-inflammatory mediators (e.g., TGF-β). In early RA, this barrier is progressively disrupted by pathological stimuli such as hypoxia and pro-inflammatory cytokines. Peripheral blood monocytes are recruited by chemokines and transmigrate across the endothelium into the sublining layer, where they rapidly polarize, under stimulation by IL-1β and ROS, into pathogenic pro-inflammatory subsets characterized by high expression of CD48, S100A12, and SPP1. These activated macrophages release abundant inflammatory mediators (e.g., TNF-α, IL-1β, and IL-6), which act in a paracrine manner to drive aberrant proliferation of fibroblast-like synoviocytes and cascading neutrophil infiltration, ultimately establishing a self-amplifying inflammatory network that drives progressive cartilage and bone destruction. Created by the authors.

In contrast, MerTK^−^CD206^−^ STMs are markedly enriched in active RA synovium. This group comprises phenotypes with distinct pathogenic mediator profiles, including CD48^+^S100A12^+^, CD48^+^SPP1^+^ and HLA^+^ISG15^+^ subsets [17,31]. The differences in spatial distribution and function between homeostasis-associated and inflammation-associated STMs are illustrated in Figure 1. Among these subsets, CD48^+^S100A12^+^ macrophages highly express alarmins (such as S100A8, S100A9 and S100A12) together with IL-8 (CXCL8). They drive the recruitment of inflammatory cells and amplify the pro-inflammatory responses of monocytes and FLS, serving as a major source of IL-1β [17,45]. CD48^+^SPP1^+^ macrophages display SPP1-associated features of tissue remodelling, migration and high glycolytic activity, and their abundance—or the expression of related genes—correlates with active synovitis and bone destruction [46,47]. HLA^+^ISG15^+^ macrophages are enriched in interferon-response genes such as ISG15, IFI6, GBP1 and STAT1, representing an IFN-responsive state within the RA synovium [17,45]. Type I IFN and IFN-γ can amplify interferon-stimulated genes, antigen presentation and inflammatory transcriptional programmes through JAK–STAT signalling, and may thereby help sustain local myeloid inflammation [48]. These subsets possess distinct inflammatory transcriptional programmes; although some of their features overlap with the conventional M1-like state, they should not be regarded as direct counterparts of classically induced M1 macrophages in vitro. The spatial distribution, representative cellular states and principal functions of synovial macrophages across healthy/remission and active RA are summarised in Figure 1.

Beyond the STMs described above, arthritis-associated osteoclastogenic macrophages (AtoMs) further connect macrophage heterogeneity to bone erosion in RA [31]. AtoMs reside mainly in RA synovial fluid and synovial tissue but are absent from peripheral blood. According to CD86 expression, they are classified into two subtypes—CD11c^+^CD86^low^ and CD11c^+^CD86^high^—that act as osteoclast precursors and participate in local CD4^+^ T cell activation and bone resorption [26,27].

In summary, the pathological role of synovial macrophages in RA does not stem from a single pro-inflammatory phenotype. It emerges instead from the collective contribution of multiple functional states, including loss of homeostasis, inflammatory recruitment, interferon responses, tissue remodelling and osteoclastogenesis. A key biological basis for the long-term persistence of their pro-inflammatory and tissue-damaging functions lies in metabolic adaptation and reprogramming in response to the aberrant local microenvironment of RA. This perspective lays the groundwork for re-examining RA pathogenesis from an immunometabolic standpoint and for developing novel therapeutic strategies.

## 4. Macrophage Metabolism in the Healthy Synovium

In the healthy synovial microenvironment, the principal role of tissue resident macrophages is not to rapidly produce large amounts of inflammatory mediators but to sustain low reactivity, high clearance capacity, and homeostatic regulation over the long term. This functional profile is supported by a corresponding metabolic program with classical M2-like features [18,49]. Energy metabolism in these cells relies primarily on mitochondrial oxidative phosphorylation (OXPHOS), with contributions from catabolic pathways such as fatty acid oxidation (FAO) and relatively low glycolytic activity [50,51]. Compared with aerobic glycolysis, OXPHOS generates ATP more efficiently and is therefore better suited to support the long-term survival of resident macrophages and their homeostatic functions, including barrier maintenance, efferocytosis, and immunoregulation [52]. FAO not only provides a stable energy supply but is also closely linked to anti-inflammatory transcriptional programs and tissue repair, helping to maintain the low-inflammatory state characteristic of healthy synovial macrophages.

Beyond meeting energy demands, this homeostatic metabolism directly shapes the immunological properties of healthy synovial macrophages. A metabolic configuration centered on OXPHOS, FAO, and amino acid metabolism restrains sustained activation of pro-inflammatory signaling, limits aberrant release of inflammatory mediators (e.g., IL-1β and TNF-α), and supports efficient clearance of apoptotic cells, matrix debris, and metabolic waste [53,54,55]. This metabolism–function coupling predisposes healthy synovial macrophages toward tissue tolerance, resolution of inflammation, and tissue turnover, providing a critical biological basis for joint homeostasis [56,57]. The healthy synovial microenvironment itself reinforces this metabolic program: balanced nutrient supply and local oxygen tension allow resident macrophages to sustain a low-inflammatory state dominated by mitochondrial respiration [58,59]. Within this context, macrophages establish stable crosstalk with synovial fibroblasts, endothelial cells, and matrix components, jointly maintaining synovial barrier integrity and the immunologically quiescent state of the joint.

In summary, the metabolic profile of healthy synovial macrophages can be characterized as a “protective metabolic state” defined by efficient energy supply, low inflammatory output, and homeostatic maintenance. This state not only underpins their tissue-protective function under physiological conditions but also offers a critical baseline for understanding how synovial macrophages, in RA, are reshaped from homeostatic guardians into persistent drivers of inflammation and tissue damage. A key feature of RA pathogenesis, then, is not simply the accumulation of more macrophages, but the progressive erosion of this homeostatic metabolic state by the pathological microenvironment, which in turn drives their functional reprogramming.

## 5. Mechanisms of Macrophage Metabolic Reprogramming in RA

Unlike resident macrophages in healthy synovium, which maintain homeostasis through OXPHOS and FAO, inflammatory macrophages in the RA synovium are defined primarily by heightened aerobic glycolysis and increased fatty acid synthesis, together with shifts in TCA cycle intermediates and amino acid metabolism. These metabolic alterations are neither a simple consequence of inflammation nor an independent upstream driver; rather, they constitute a bidirectional feedback loop between the inflammatory microenvironment and macrophage function. Hypoxia, inflammatory cytokines and ACPA-related immune complexes can dismantle the homeostatic programme of resident macrophages and exert sustained upstream pressure that favours inflammatory phenotypes and metabolic adaptation [17,18,19]. The lactate, succinate, lipid mediators and metabolic enzyme activities thereby generated, in turn, reinforce cytokine production and tissue-destructive functions.

### 5.1. Glucose Metabolism

#### 5.1.1. Increased Glucose Uptake and the Establishment of the Warburg Effect in RA Macrophages

**Figure 2:**
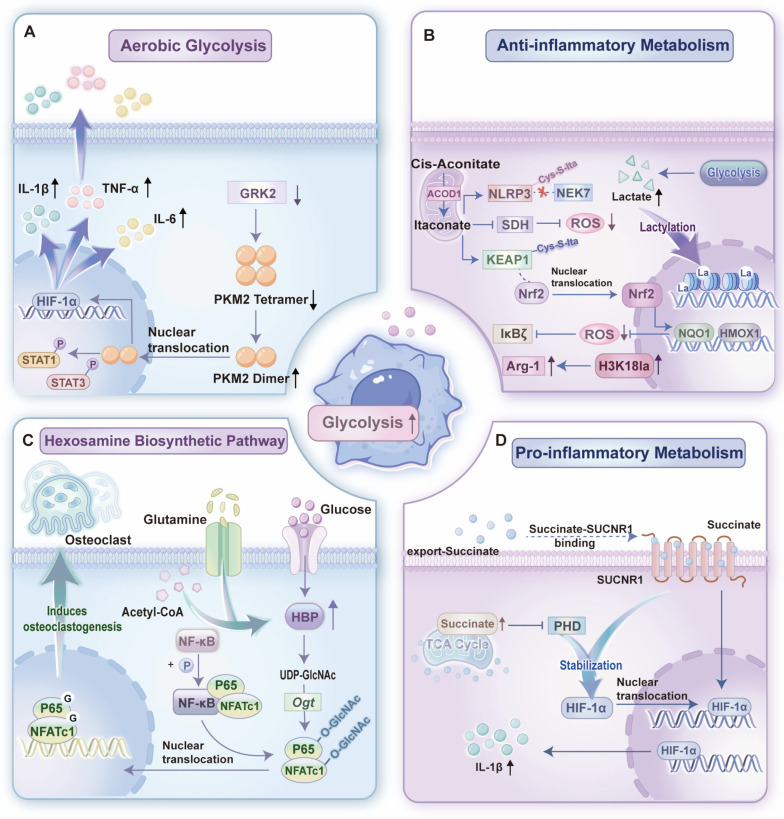
Multidimensional regulation of glucose metabolic reprogramming in RA synovial macrophages. (**A**) GRK2 downregulation relieves post-translational constraints on PKM2, thereby promoting its nuclear translocation and functional cooperation with HIF-1α and STAT1/3 to establish a self-reinforcing inflammatory circuit. (**B**) In parallel, this glycolytic shift disrupts the TCA cycle and drives the accumulation of immunomodulatory metabolites. Itaconate and lactate cooperatively modulate inflammation through distinct mechanisms: itaconate inhibits NLRP3 activation and induces the KEAP1/NRF2 antioxidant program, while lactate mediates histone H3K18 lactylation. (**C**) Simultaneously, enhanced flux through the HBP increases UDP-GlcNAc production, fueling OGT-mediated O-GlcNAcylation of NF-κB p65 and NFATc1. This modification stabilizes these master transcription factors and promotes their nuclear translocation, ultimately driving osteoclast differentiation and bone resorption. (**D**) Disruption of the TCA cycle drives succinate accumulation in RA synovial macrophages. Intracellular succinate inhibits PHD activity, resulting in HIF-1α stabilization and nuclear translocation, thereby promoting IL-1β production. Meanwhile, extracellular succinate engages SUCNR1 signaling to further amplify pro-inflammatory metabolic responses. Created by the authors.

Regarding glucose metabolism, enhanced glucose uptake marks the initial step of metabolic reprogramming. The local hypoxic microenvironment of the RA synovium, combined with pro-inflammatory signals such as TNF-α, IL-1β, and IL-6, synergistically induces the upregulation of glucose transporter 1 (GLUT1) on the macrophage surface, thereby facilitating glucose influx and intracellular utilization [60]. Following this increased uptake, macrophages no longer primarily rely on the entry of pyruvate into the mitochondria for complete oxidation. Instead, they prioritize rapid ATP generation via glycolysis, converting substantial amounts of pyruvate into lactate to establish the classic Warburg effect [61,62]. In this state, even with adequate oxygen supply, cytosolic pyruvate largely bypasses the mitochondrial TCA cycle and is instead predominantly reduced to lactate by lactate dehydrogenase (LDH) [63,64]. Although this metabolic shift yields less energy per glucose molecule, its rapid kinetics are better suited to meet the heightened macrophage demands for energy, biosynthetic precursors, and swift effector responses during inflammation—factors closely associated with inflammatory activity, synovial invasion, and bone destruction. Consequently, this metabolic transition serves as a crucial foundation for sustaining the pro-inflammatory activation of RA synovial macrophages. The major pathways linking glucose metabolic reprogramming to macrophage function and osteoclastogenesis are summarised in Figure 2.

#### 5.1.2. Key Glycolytic Enzymes and Their Post-Translational Modification Networks

In RA macrophages, the expression of key glycolytic enzymes—such as pyruvate kinase M2 (PKM2), hexokinase 2 (HK2), and 6-phosphofructo-2-kinase/fructose-2,6-biphosphatase 3 (PFKFB3)—is significantly upregulated. Notably, these enzymes act as more than mere catalysts that directly amplify cellular glycolytic flux; they also exhibit non-metabolic “moonlighting functions”. By translocating to the nucleus and directly interacting with core transcription factors such as HIF-1α and STAT, they regulate the transcription of pro-inflammatory target genes, including IL-1β, TNF-α, and IL-6. Studies utilizing the collagen-induced arthritis (CIA) mouse model have demonstrated that aberrantly active PKM2 potently activates STAT1/STAT3 signaling, inducing M1 macrophage polarization and subsequently accelerating synovial inflammation and articular bone destruction [65]. Furthermore, in a TNF-transgenic arthritis mouse model, overexpressed PFKFB3 drives similar M1 polarization, exacerbating joint pathology. Mechanistically, PFKFB3 achieves this not only by enhancing glycolysis but also by directly binding to glutamate dehydrogenase 1; this interaction inhibits glutamate dehydrogenase (GDH) activity, leading to the depletion of α-ketoglutarate. Conversely, macrophage-specific conditional knockout of PFKFB3 restores α-ketoglutarate levels, significantly ameliorating the clinical symptoms of arthritis and joint destruction [66]. Collectively, these findings highlight that key glycolytic enzymes are not merely passive executors of metabolic reprogramming, but rather serve as crucial regulatory hubs within the inflammatory transcriptional network.

Furthermore, the inflammatory microenvironment in RA finely orchestrates macrophage glycolysis through specific post-translational modification networks, with the GRK2-PKM2 axis being particularly crucial. Under physiological conditions, G protein-coupled receptor kinase 2 (GRK2) mediates the phosphorylation of PKM2 at Ser406 and promotes its desuccinylation at Lys433, thereby restricting aberrant glycolytic activation [67,68]. Conversely, in pathological RA macrophages, downregulated GRK2 expression relieves these structural constraints on PKM2. This relief promotes PKM2 dimerization and subsequent nuclear translocation, where it functions as a transcriptional coactivator to exacerbate glycolysis [69]. Crucially, nuclear PKM2 cooperates with HIF-1α and STAT3 to upregulate key glycolytic enzymes, including HK2 and PFKFB3. This reinforces a self-amplifying pro-inflammatory feedback loop and promotes the substantial accumulation of lactate and succinate. These accumulated metabolites further stimulate macrophages to release inflammatory cytokines, including TNF-α, IL-6, and IL-1β [70,71], effectively locking the synovial microenvironment into a chronically hyperactive, metabolic-inflammatory coupled state. Consequently, the aberrant expression of key enzymes, combined with the post-translational modification-mediated establishment of the PKM2-HIF-1α positive feedback loop, collectively constitutes the core molecular foundation that sustains and amplifies the pro-inflammatory glycolytic program in RA macrophages.

#### 5.1.3. Metabolic Intermediate-Mediated Immunometabolic Signaling

Beyond the upregulation of the main glycolytic pathway, intermediates of glucose metabolism and of the disrupted tricarboxylic acid (TCA) cycle, particularly lactate, succinate, and itaconate, accumulate substantially within the RA synovial microenvironment [72]. Far from acting merely as energy intermediates, these metabolites function as central immunometabolic signaling molecules and epigenetic regulators, exerting multifaceted control over macrophage fate decisions [73,74,75,76].

In lactate metabolism, Zhang and colleagues first demonstrated, in mouse bone marrow-derived macrophages, that lactate can directly serve as a substrate to mediate histone lysine lactylation (e.g., H3K18la). This epigenetic modification selectively activates transcription of anti-inflammatory genes (e.g., Arg1), thereby driving the transition of macrophages from a pro-inflammatory to a tissue-reparative phenotype [77]. Subsequent clinical studies have shown that several lactate metabolism-related genes, including KCNN4 and SLC25A4, are aberrantly expressed in patients with RA and contribute to macrophage functional remodeling [78]. Notably, lactate exerts cell type-specific effects on RA-FLSs and macrophages: in RA-FLSs, lactate is taken up via the SLC16A3 transporter and acts as a pro-migratory signal, enhancing invasiveness and promoting IL-6 secretion; in macrophages, by contrast, lactylation acts as an immunological “brake”, driving M2 polarization while suppressing migration, glycolytic flux, and IL-6 secretion [79]. These findings suggest that lactate carries dual—both pathogenic and disease-restraining—properties in RA, with its net effect dictated by cell type and local microenvironmental context.

Unlike lactate, succinate functions more unequivocally as a central pro-inflammatory metabolite in RA. Under inflammatory stimulation, macrophages markedly upregulate aconitate decarboxylase 1 (ACOD1/IRG1), which catalyzes the conversion of cis-aconitate to itaconate, thereby disrupting the TCA cycle and causing upstream accumulation of citrate and succinate [72,80]. Accumulated succinate, in turn, acts as a key pro-inflammatory driver of RA synovitis. Intracellularly, it stabilizes HIF-1α by competitively inhibiting prolyl hydroxylase domain (PHD) enzymes [81,82]. More detrimentally, excess intracellular succinate is exported to the extracellular space, where it engages its cognate G protein-coupled receptor SUCNR1 in an autocrine and paracrine manner, further stabilizing HIF-1α and amplifying IL1B transcription to generate a self-reinforcing inflammatory loop [83]. As a master regulator of the glycolytic network, activated HIF-1α upregulates the expression of multiple glycolytic enzymes and glucose transporters, thereby consolidating the pathogenic glycolytic state [71,84].

In contrast, itaconate, which is generated by ACOD1 (encoded by IRG1, immune-responsive gene 1), serves as an endogenous negative regulator of innate immune metabolism, exerting predominantly tissue-protective and anti-inflammatory effects. Clinical studies have shown that itaconate levels in plasma and synovial fluid of patients with RA are significantly elevated during the early active phase and inversely correlate with DAS28 scores and inflammatory markers such as TNF-α and IL-6, suggesting that itaconate may act as an endogenous metabolite mobilized to restrain inflammatory expansion [85]. Mechanistically, in the context of oxidative stress, itaconate alkylates key cysteine residues of KEAP1, releasing Nrf2 from KEAP1-mediated ubiquitination and enabling its nuclear translocation to activate antioxidant genes, including HMOX1 and NQO1; this in turn reduces ROS burden and suppresses IκBζ-dependent transcription of pro-inflammatory genes [86]. At the inflammasome level, itaconate modifies cysteine 548 of NLRP3, disrupting its interaction with NEK7 and thereby directly inhibiting NLRP3 inflammasome assembly and the maturation and release of IL-1β and IL-18 [87]. More recently, several high-impact studies have established that suppression of osteoclast differentiation is a key mechanism through which itaconate attenuates bone erosion in RA. Its cell-permeable esterified derivative, 4-octyl itaconate (4-OI), selectively inhibits the DNA demethylase TET2, epigenetically blocking osteoclastogenesis and progressive bone destruction in the RA microenvironment [88]. The ACOD1–itaconate axis also competitively inhibits succinate dehydrogenase (SDH), curbing mitochondrial ROS production and HIF-1α-driven aerobic glycolysis, and thereby directly suppressing osteoclast differentiation [89]. Overall, lactate, succinate, and itaconate together constitute a “pro-inflammatory–braking” metabolic signaling network in the RA synovium, whose dynamic balance largely dictates the inflammatory fate of macrophages and the trajectory of bone destruction.

#### 5.1.4. The Hexosamine Biosynthesis Pathway and O-GlcNAcylation

Beyond canonical energy-generating pathways, the hexosamine biosynthesis pathway (HBP) serves as a central hub linking nutrient sensing to signal transduction, playing an indispensable role in shaping glucose metabolic reprogramming in macrophages. The HBP integrates inputs from glucose, glutamine, and acetyl-CoA to synthesize the key nucleotide-sugar donor uridine diphosphate N-acetylglucosamine (UDP-GlcNAc) [90]. UDP-GlcNAc then serves as the sole substrate for O-GlcNAc transferase (OGT), which catalyzes O-GlcNAcylation of target proteins [91]. Within the pathological microenvironment of RA, aberrantly elevated HBP flux profoundly remodels the epigenetic and transcriptional landscape of macrophages. Notably, in osteoclast precursors residing at the synovium–bone interface, heightened O-GlcNAcylation acts as a critical molecular switch driving terminal differentiation into osteoclasts [92]. Mechanistic studies have shown that O-GlcNAcylation of NF-κB p65 and NFATc1, the master regulator of osteoclastogenesis, is a metabolic prerequisite for stabilizing their protein conformation and promoting nuclear translocation [93]. Targeting this metabolism–post-translational modification axis holds considerable therapeutic promise. shRNA-mediated knockdown of Ogt phenocopies the inhibitory effects of the OGT inhibitor OSMI-1 [94]. Together, these pharmacological and genetic findings suggest that targeting the O-GlcNAcylation network during macrophage-to-osteoclast commitment may offer a translationally valuable strategy for curbing progressive bone destruction in RA and other diseases driven by aberrant osteoclast activation.

In summary, the essence of macrophage glucose metabolic reprogramming in RA is a transition from “oxidative utilization that sustains synovial homeostasis” to “rapid mobilization in service of inflammatory amplification and tissue destruction”. This transition begins with enhanced GLUT1-mediated glucose uptake and the establishment of the Warburg effect, is progressively amplified through key glycolytic enzymes such as PKM2, HK2, and PFKFB3 and their post-translational modification networks, and is further stabilized by immunometabolic signaling driven by intermediates including lactate, succinate, and itaconate, which collectively reinforce the pathogenic state of macrophages. In parallel, the HBP–O-GlcNAc axis directly couples glucose metabolic dysregulation to osteoclast differentiation and bone erosion. Targeting aberrant glucose metabolic nodes such as GLUT1, HIF-1α, and key glycolytic enzymes has therefore emerged as one of the most representative strategies in RA metabolic intervention research.

### 5.2. Lipid Metabolism

#### 5.2.1. Lipid Uptake, Lipid Droplet Formation, and De Novo Fatty Acid Synthesis

In contrast to the FAO-dominated metabolism of homeostatic M2 macrophages in the healthy synovium, M1 macrophages in the RA inflammatory microenvironment shift toward lipid synthesis and accumulation [95,96]. Clinical studies have shown that phospholipid, triglyceride, and cholesterol levels are markedly altered in the serum and synovial fluid of patients with RA [97,98]. Within this lipid-enriched milieu, macrophages upregulate the uptake of exogenous free fatty acids and esterify them into triglycerides, which are stored in lipid droplets and serve both as energy reserves and as a precursor pool for the biosynthesis of lipid mediators of inflammation, such as prostaglandins [99]. This lipid reprogramming is tightly coupled to glucose metabolism: citrate accumulating in the disrupted TCA cycle is exported to the cytosol and cleaved into acetyl-CoA, providing the carbon source for fatty acid synthesis [100], while the activated pentose phosphate pathway (PPP) supplies abundant NADPH as reducing equivalents [101]. Catalyzed by fatty acid synthase, acetyl-CoA and NADPH together drive robust de novo synthesis of fatty acids [102]. These newly synthesized lipids not only provide essential building blocks for the membrane expansion required during rapid macrophage activation, but also function as key lipid signaling molecules that sustain M1 polarization. Lipid metabolic dysregulation in RA is therefore not a passive byproduct of inflammation but an active driver in sustaining and amplifying the inflammatory microenvironment.

#### 5.2.2. Glucocorticoid Metabolism

Glucocorticoids are cholesterol-derived steroid hormones, and their local metabolism constitutes an important endogenous pathway through which macrophages regulate inflammation. 11β-hydroxysteroid dehydrogenase (11β-HSD) is the central enzyme system of this pathway: 11β-HSD1 catalyses the local conversion of inactive cortisone into active cortisol, whereas 11β-HSD2 mediates the inactivation of cortisol [103]. Recent studies show that HSD11B1, the gene encoding 11β-HSD1, is enriched predominantly in the SPP1^+^ STMs associated with active RA, while remaining comparatively low in most other STM subsets [104]. In macrophages differentiated in vitro from healthy-donor monocytes, IFN-γ and TNF-α induce HSD11B1 expression and 11β-HSD1 activity, enhancing the conversion of cortisone to cortisol and thereby suppressing the release of TNF-α and IL-6 [105]. Consistent with this, in myeloid-specific HSD11B1-knockout mice, loss of macrophage 11β-HSD1 aggravates the impaired resolution of inflammation in experimental arthritis and promotes pathological angiogenesis [105]. Together, these findings suggest that the distribution of 11β-HSD1 across macrophages may be state-dependent, and that local cortisol regeneration in SPP1^+^ STMs may serve as an inflammation-induced compensatory negative-feedback mechanism.

#### 5.2.3. Arachidonic Acid Metabolism

Within the broader landscape of lipid metabolic reprogramming, the metabolic cascade of arachidonic acid (AA), a polyunsaturated fatty acid, occupies a central position in RA pathogenesis. Under physiological conditions, AA is stored in acylated form within the phospholipid bilayer of the cell membrane. In RA, locally enriched cytokines such as TNF-α and IL-1β robustly and persistently activate the MAPK pathway, which in turn drives phosphorylation and membrane translocation of cytosolic phospholipase A2 (cPLA2), liberating free AA from membrane phospholipids [106,107,108]. Free AA is then converted by cyclooxygenases (COX), lipoxygenases (LOX), and cytochrome P450 (CYP450) enzymes into a family of bioactive lipid mediators collectively termed eicosanoids [109]. Targeted lipidomics and in situ histological analyses have revealed marked elevations of downstream AA metabolites in the serum, synovial fluid, and synovial tissue of patients with RA, including prostaglandin E2 (PGE2), thromboxane A2 (TXA2), leukotriene B4 (LTB4), and the full spectrum of hydroxyeicosatetraenoic acids (HETEs), namely 5-, 8-, 12-, 15-, and 12(S)-HETE [98]. These pro-inflammatory lipids act as potent paracrine and autocrine signals that sustain macrophage recruitment, amplify the inflammatory cascade, and drive progressive joint destruction. Lipid metabolic reprogramming centered on the AA cascade thus represents both a defining metabolic feature of the RA synovial microenvironment and a fundamental driver of disease progression.

#### 5.2.4. The PGE2–EP4–GRK2 Axis

**Figure 3:**
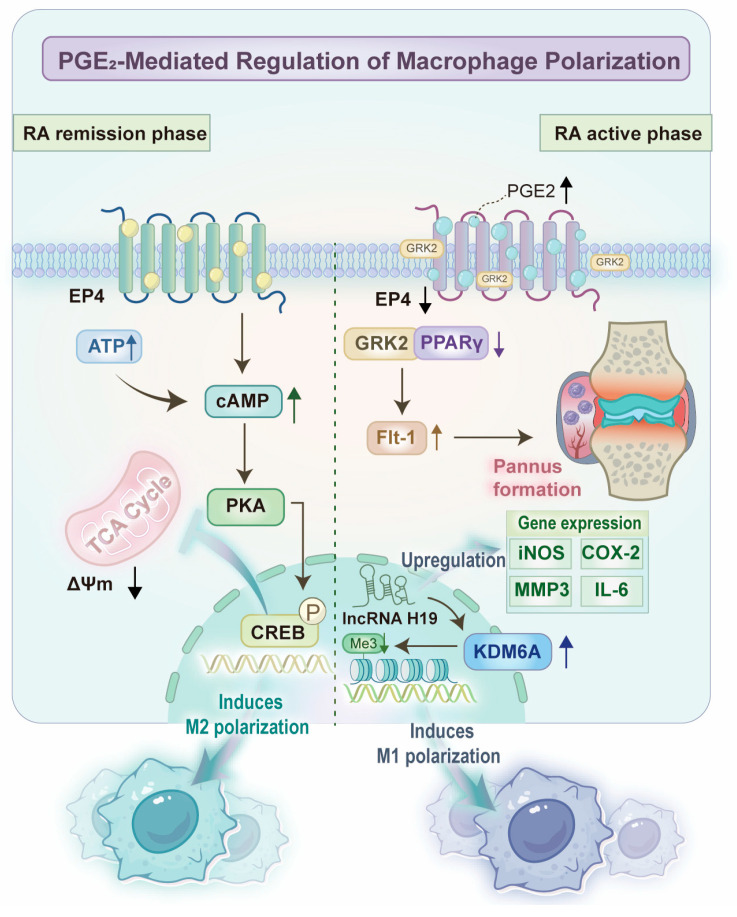
The PGE2–EP4 axis exhibits bidirectional regulation in RA. During RA remission, PGE2 engages EP4 to activate the cAMP–PKA–CREB anti-inflammatory axis, suppressing the TCA cycle and lowering the mitochondrial membrane potential (ΔΨm), thereby driving M2 polarization and FAO-dominated metabolism. In active RA, sustained high concentrations of PGE2 promote GRK2-mediated phosphorylation, internalization, and homologous desensitization of EP4. On the one hand, the lncRNA H19–KDM6A demethylation axis upregulates pro-inflammatory genes including iNOS, COX-2, MMP3, and IL-6. On the other hand, depletion of the cytosolic GRK2 pool abolishes its stabilizing effect on PPARγ, resulting in uncontrolled Flt-1 transcription and transendothelial infiltration of monocytes and macrophages, which together drive pannus formation. Created by the authors.

As the principal terminal product of the COX-mediated lipid cascade, PGE2 reshapes macrophage polarization in a highly context-dependent and bidirectional manner. The context-dependent effects of PGE2–EP4–GRK2 signalling on macrophage function are illustrated in Figure 3. During RA remission and physiological tissue repair, PGE2 exerts anti-inflammatory effects by engaging EP4 receptors on macrophages. This activates the cAMP–PKA–CREB pathway and suppresses inflammatory responses. This signalling cascade also induces metabolic remodelling. It lowers the mitochondrial membrane potential (ΔΨm) and suppresses both the TCA cycle and the malate–aspartate shuttle, thereby reinforcing an M2-like metabolic phenotype [110,111].

However, in the pathological microenvironment of active RA, this physiological “brake” is overturned by receptor desensitization and epigenetic rewiring, redirecting PGE2 toward driving M1 polarization. In the CIA model, prolonged exposure of the synovium to high local concentrations of PGE2 aberrantly activates G protein-coupled receptor kinase 2 (GRK2) in macrophages. Activated GRK2 hyperphosphorylates EP4, leading to homologous receptor desensitization and endocytic degradation. This attenuates signals associated with M2 polarization and instead favours an M1-like phenotype [112]. Under these conditions, PGE2–EP4 engagement stimulates macrophages to release pro-inflammatory cytokines such as IL-6 and IL-1β, exacerbating bone resorption, bone loss, and joint destruction [113]. In this process, large amounts of GRK2 become sequestered at the plasma membrane to phosphorylate EP4, severely depleting the cytosolic GRK2 pool [112]. Under physiological conditions, cytosolic GRK2 interacts with peroxisome proliferator-activated receptor γ (PPARγ) and sustains its transcriptional activity. PPARγ subsequently suppresses expression of the pro-angiogenic receptor FMS-like tyrosine kinase 1 (Flt-1/VEGFR-1), thereby restraining aberrant synovial neovascularization. Depletion of the cytosolic GRK2 pool reduces PPARγ activity and permits Flt-1 upregulation. This promotes the infiltration of monocytes and macrophages into the joint and contributes to pannus formation and expansion [114]. Aberrant GRK2 activation therefore does not act on a single target. EP4 hyperphosphorylation may therefore act as a central node linking two mutually reinforcing pathological processes. The first is macrophage polarization imbalance, which promotes cytokine-mediated bone damage. The second is pannus formation, which facilitates sustained immune-cell infiltration. Together, these processes establish a self-amplifying circuit that contributes to progressive joint destruction.

In addition, at the epigenetic level, aberrantly elevated expression of the long non-coding RNA (lncRNA) H19 upregulates the histone demethylase KDM6A, promoting M1 polarization of macrophages and increasing the expression of iNOS, IL-6, matrix metalloproteinases (MMP3/13), and COX-2. This in turn promotes further synthesis and release of PGE2, establishing a self-sustaining feedback loop between lipid metabolism and inflammatory epigenetic regulation in the local synovium [115]. PGE2 is therefore neither exclusively pro-inflammatory nor uniformly anti-inflammatory. Its immunometabolic effects depend on receptor availability, the pre-existing macrophage state, and the surrounding signalling network. Based on this mechanism, targeting GRK2 in macrophages to restore EP4 receptor membrane localization and sensitivity may interrupt the pathogenic PGE2 loop and represents a promising strategy to redirect macrophages toward the M2 phenotype.

#### 5.2.5. The LTB4-BLT1 Axis

**Figure 4:**
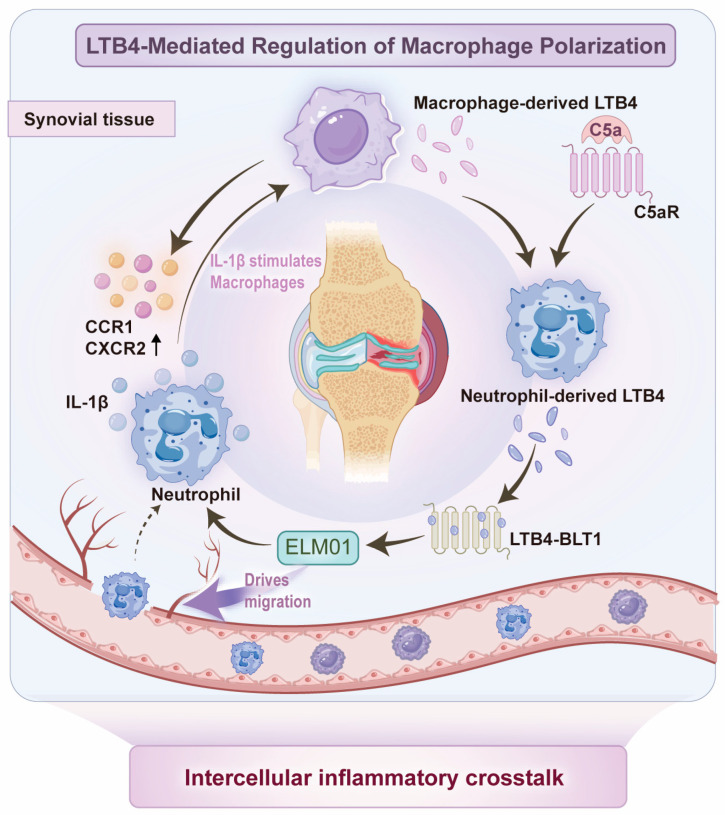
Trans-cellular inflammatory circuit mediated by LTB4. Macrophage-derived LTB4 and complement-cascade-derived C5a independently and synergistically activate neutrophils through BLT1 and C5aR, respectively. Activated neutrophils synthesize additional LTB4 via their own 5-LOX, establishing an autocrine amplification pool, and enhance transendothelial migration through the BLT1–ELMO1 axis. Concurrently, neutrophils release IL-1β, which feeds back to stimulate macrophages to secrete CCR1- and CXCR2-targeting chemokines. Created by the authors.

Beyond the COX–PGE2 axis, LTB4, the principal derivative of the LOX-mediated lipid network, is recognized as one of the most potent endogenous chemotactic lipids driving the pro-inflammatory microenvironment. The LTB4-driven macrophage–neutrophil inflammatory circuit is illustrated in Figure 4. In RA pathogenesis, the central pathogenic role of LTB4 lies in its coupling with the high-affinity receptor BLT1, which mediates cascade recruitment of neutrophils [116]. Classical studies have shown that the LTB4–BLT1 axis acts as a “pioneer signal” that drives directed transendothelial migration of neutrophils into the joint cavity, and that loss or blockade of BLT1 markedly attenuates the severity of arthritis [117]. Within the synovial microenvironment, macrophage-derived LTB4 acts synergistically with activated complement (C5a–C5aR) to stimulate further lipid synthesis in neutrophils, which release large amounts of LTB4 to form a powerful autocrine amplification pool. Meanwhile, LTB4 engages BLT1 to activate the downstream migration signal ELMO1, enhancing neutrophil mobility and amplifying chemotactic and inflammatory responses [118,119]. More importantly, this lipid-driven chemotactic process is not unidirectional. Activated neutrophils release bursts of pro-inflammatory mediators such as IL-1β, which in turn stimulate macrophages to produce CCR1- and CXCR2-targeting chemokines, establishing a lipid–chemokine positive feedback loop that sustains chronic inflammation and tissue destruction [120].

In summary, macrophage lipid metabolic reprogramming in RA represents a shift from an FAO-based mode that sustains immune homeostasis and tissue repair toward a pro-inflammatory program characterized by enhanced lipid uptake, lipid droplet accumulation, de novo fatty acid synthesis, and activation of the AA cascade. This program not only provides the substrate basis for the sustained generation of lipid mediators such as prostaglandins and leukotrienes, but also couples, through the PGE2–EP4–GRK2 and LTB4–BLT1 axes respectively, macrophage polarization imbalance, pannus formation, and cascade recruitment of neutrophils, thereby collectively driving the amplification of synovial inflammation and the progression of joint destruction in RA.

### 5.3. Amino Acid Metabolism

#### 5.3.1. Glutamine Metabolism

In terms of amino acid metabolism, RA synovial macrophages display markedly enhanced glutaminolysis, characterized by increased glutamine uptake through the SLC1A5/ASCT2 transporter, elevated GLS-dependent deamidation flux, and intracellular accumulation of α-ketoglutarate (α-KG) and glutamate [11,121]. Unlike the balanced amino acid utilization observed in resting macrophages, this metabolic shift enables continuous supply of the carbon skeletons, nitrogen sources, and signaling substrates required to sustain inflammatory activation, thereby providing metabolic support for the pro-inflammatory phenotype. This metabolism-inflammation coupling is not an isolated intracellular event but amplifies inflammation through crosstalk among multiple immune cell types. On the one hand, inflammatory RA-FLSs upregulate, via paracrine signaling, SLC1A5-mediated glutamine uptake and the expression of pro-inflammatory genes (Nos2, Tnf, and Il1b) in macrophages, prolonging their pro-inflammatory survival [122]. On the other hand, elevated GLS1 activity stabilizes the M1 phenotype and promotes Th17 cell differentiation through an mTORC1-dependent pathway, while IL-17 feedback further upregulates GLS1 expression in FLSs. This circuit sustains synovial inflammation and prevents its spontaneous resolution [123,124]. Enhanced glutaminolysis therefore is not simply a metabolic feature of RA synovial macrophages but serves as a key metabolic hub linking innate and adaptive immunity and sustaining chronic inflammation.

#### 5.3.2. Arginine Metabolism

**Figure 5:**
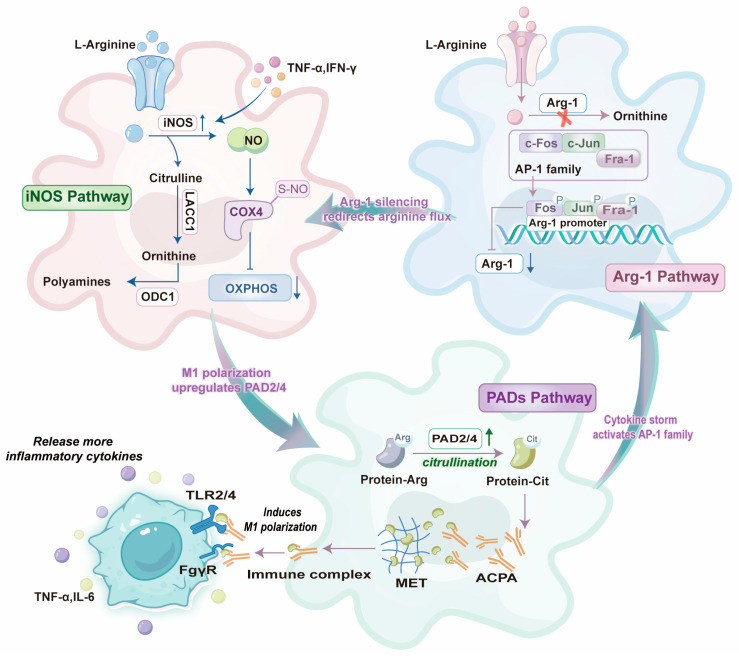
Arginine metabolic reprogramming and the self-reinforcing pathogenic loop in RA macrophages. Within the RA synovial inflammatory microenvironment, L-arginine metabolism in macrophages is reshaped into three interconnected pathogenic branches. iNOS pathway: Under TNF-α and IFN-γ stimulation, iNOS is markedly upregulated and catalyzes the conversion of L-arginine into NO and citrulline. NO inhibits COX4 activity through S-nitrosylation, blocking OXPHOS. LACC1 further metabolizes citrulline through the ornithine–ODC1 route to generate polyamines, linking the pro-inflammatory NO pathway with the reparative polyamine pathway. Arg-1 pathway: The AP-1 transcription factors c-Fos, c-Jun, and Fra-1 bind to the TRE site of the Arg1 promoter and silence its transcription, redirecting the arginine substrate toward the iNOS pathway. PADs pathway: PAD2 and PAD4 convert arginine residues in peptide chains into citrulline residues. The resulting citrullinated self-antigens are released through METs and form immune complexes with ACPAs, which cross-link TLR2/4 and FcγRs on neighboring macrophages and trigger the cascade release of pro-inflammatory cytokines such as TNF-α and IL-6. Created by the authors.

L-arginine sits at a key intersection of cellular metabolism and immune regulation, and its metabolic flux undergoes profound pathological reprogramming in RA synovial macrophages. The major arginine-related metabolic and citrullination pathways in RA macrophages are summarised in Figure 5. This reprogramming features three distinct branches: iNOS-mediated cytotoxic oxidation, loss of the reparative function of arginase-1 (Arg-1), and pathogenic post-translational modifications driven by peptidylarginine deiminases (PADs). Within synovial macrophages, these three branches converge and reinforce one another, locking the cells into a self-amplifying vicious cycle of dysregulated metabolism, autoimmunity, and chronic inflammation that resists spontaneous resolution [125,126].

iNOS-Mediated Toxic Oxidation. Under sustained stimulation by pro-inflammatory cascades dominated by IFN-γ and TNF-α, inducible nitric oxide synthase (iNOS) is robustly upregulated within macrophages. This heightened iNOS activity rapidly depletes intracellular L-arginine, catalyzing its conversion into free citrulline and generating high concentrations of nitric oxide (NO) [127,128]. As a potent gaseous signaling molecule, NO directly mediates the S-nitrosylation of mitochondrial electron transport chain (ETC) complex IV (COX4), inhibiting its enzymatic activity and stalling OXPHOS flux. This metabolic blockade forces macrophages to rely on inefficient yet rapid aerobic glycolysis, thereby metabolically stabilizing their pro-inflammatory phenotype. Traditionally, the iNOS and Arg-1 pathways have been viewed as strictly antagonistic, competing for the same L-arginine pool. However, recent studies have revealed that the key metabolic enzyme LACC1 (also known as FAMIN) can convert iNOS-derived citrulline into ornithine. This ornithine is subsequently metabolized into polyamines by ornithine decarboxylase 1 (ODC1), effectively coupling the pro-inflammatory NO pathway with the reparative polyamine pathway [129]. This compensatory mechanism carries profound clinical implications. Genetic and pathological evidence indicates that loss-of-function mutations in LACC1 lead to deficient polyamine synthesis in macrophages, thereby disabling a critical endogenous immune brake. The resulting unbridled inflammatory cascade drives the pathogenesis of severe systemic autoimmune diseases, including juvenile idiopathic arthritis (JIA) and RA [130,131].

Loss of the reparative function of Arg-1. In contrast, the Arg-1 pathway is markedly suppressed during active RA. Under physiological conditions, Arg-1 serves as a hallmark metabolic enzyme of M2 macrophages, catalyzing the conversion of L-arginine into ornithine and urea. Ornithine is then used to synthesize polyamines and proline, which provide essential substrates for collagen deposition, synovial tissue remodeling, and the resolution of inflammation [128,132,133]. In the RA synovial microenvironment, however, this reparative axis is shut down through transcriptional reprogramming. Recent studies have shown that members of the AP-1 transcription factor complex, including Fra-1, c-Fos, and c-Jun, are aberrantly overexpressed in RA macrophages and bind directly to the core promoter region of Arg1 to silence its transcription [121,134,135]. This transcriptional silencing sharply reduces Arg-1 enzymatic activity and redirects the limited intracellular arginine pool toward the iNOS pathway, fueling sustained NO synthesis and reinforcing the M1 pro-inflammatory phenotype [136]. Targeting this transcriptional–metabolic imbalance holds considerable therapeutic promise. Metabolic flux experiments have shown that supplementation with exogenous L-arginine, through substrate saturation, can outcompete iNOS, restore the anti-inflammatory flux of Arg-1, and effectively attenuate arthritis progression in animal models [134]. Accordingly, Fra-1 has emerged as a promising therapeutic target in RA. The development of selective Fra-1 antagonists may relieve transcriptional repression of Arg1 at its source, restore the endogenous reparative capacity of macrophages, and open a translationally valuable avenue for immunometabolic therapy in RA.

Pathogenic post-translational modifications driven by PADs. Among the branches of arginine metabolism, PAD-mediated protein citrullination is the most RA-specific pathogenic modification and plays a central role in the synovial microenvironment. Unlike other arginine-utilizing pathways, this process does not consume free cytosolic arginine but instead converts arginine residues within protein peptide chains into citrulline residues. In RA synovial macrophages, the isoenzymes PAD2 and PAD4 are constitutively overexpressed [137]. Their aberrant activity generates large amounts of citrullinated self-antigens, which drive the production of ACPAs and trigger a joint-specific autoimmune response [138,139]. Beyond generating self-antigens, citrullination also acts as a microenvironmental signal that reshapes macrophage polarization. Citrullinated fibrinogen in the extracellular matrix drives macrophages toward an M1 phenotype and induces secretion of inflammatory mediators such as IL-18, IL-13, and CCL22. This pro-inflammatory polarization, in turn, further upregulates PAD2 transcription, establishing a positive-feedback loop between protein citrullination and inflammatory amplification [140]. Highly activated macrophages also rely on the catalytic activity of PAD2 and PAD4 to generate macrophage extracellular traps (METs). During active RA, excessive METs carrying citrullinated proteins bind to ACPAs to form highly pathogenic immune complexes, which cross-link Fcγ receptors (FcγRs) and Toll-like receptors 2/4 (TLR2/4) on neighboring macrophages, recruiting and activating additional macrophages and driving destructive cytokine storms of TNF-α and IL-6 [141,142]. This citrullination-driven network is further sustained by upstream systemic danger signals. Recent studies have shown that aminoacyl-tRNA synthetases (aaRSs), aberrantly secreted into the synovial fluid and serum of patients with RA, act as potent alarmins that stimulate macrophages to produce IL-6 and IL-1β and promote PAD4 release. This newly identified alarmin–PAD4 axis transcends the physical limits of the plasma membrane and propels macrophage-mediated citrullination-driven inflammation toward unrestrained amplification [143].

In summary, amino acid metabolic reprogramming in the RA synovium is not driven by a single pathway but by the convergence of enhanced glutaminolysis, redirected arginine metabolism, and PAD-mediated aberrant citrullination, which together form a self-reinforcing pathogenic network. Within this network, Fra-1-mediated suppression of Arg-1 redirects arginine toward the iNOS pathway, where iNOS continuously consumes arginine to sustain the pro-inflammatory M1 phenotype. In parallel, immune complexes formed through the PAD2/4–ACPA pathway act as potent transmembrane agonists that further upregulate iNOS expression. This closed pathological loop deprives local synovitis of any opportunity for spontaneous resolution. Beyond reshaping macrophage polarization, arginine metabolic imbalance also directly affects joint bone homeostasis. Terminal differentiation of osteoclast precursors, which are essentially a subset of synovium-resident macrophages, depends on the sensing of specific metabolic substrates. Recent pharmacological evidence has shown that supplementation with exogenous L-arginine reverses TNF-α-induced mitochondrial damage in osteoclast precursors, restoring OXPHOS flux and ATP production, and thereby suppressing osteoclast differentiation and bone resorption [144]. These findings suggest that local arginine depletion in the RA synovial microenvironment not only amplifies the macrophage-driven inflammatory storm but also releases the metabolic brake on osteoclasts, jointly driving irreversible and progressive cartilage and bone erosion. The essence of amino acid metabolic reprogramming in RA is therefore the conversion of amino acid utilization, originally dedicated to cellular homeostasis, tissue repair, and immune balance, into a pathological program that supports sustained macrophage inflammation, ACPA-driven autoimmune amplification, and aberrant osteoclastogenesis. From a therapeutic perspective, targeting key nodes such as the SLC1A5/GLS1, LACC1, Fra-1/Arg-1, and PAD2/PAD4 axes may provide new mechanistic foundations and translational directions for immunometabolic intervention in RA.

## 6. Therapeutic Modulation of Macrophage Metabolism in RA

### 6.1. Repurposed Anti-Rheumatic Drugs with Metabolic Effects

The recognition that aberrant macrophage metabolism contributes to persistent RA inflammation and to variable treatment responses has prompted a re-examination of how marketed antirheumatic drugs work. A growing body of evidence points beyond their classical immunomodulatory effects. Glucocorticoids and methotrexate can also directly reshape the mitochondrial and one-carbon metabolic programmes of macrophages. This offers a clinically relevant mechanistic rationale for evaluating metabolic reprogramming as a therapeutic target in RA.

Glucocorticoids (GCs) are a first-line treatment for RA recommended by the EULAR guidelines [145]. Their classical action has long been attributed to transcriptional repression mediated by the GC receptor (GR). Recent work, however, has uncovered a non-genomic metabolic mechanism. In the cytoplasm, GR can bind directly to a regulatory subunit of pyruvate dehydrogenase (PDH). This induces the translocation of PDH into mitochondria and enhances its activity. The result is expanded TCA cycle flux and a sustained supply of cis-aconitate, the substrate of ACOD1, which upregulates synthesis of the anti-inflammatory metabolite itaconate [146]. In the K/BxN serum-transfer arthritis model, loss of ACOD1 largely abolishes the anti-arthritic effects of GCs [146]. This suggests that reprogramming of the TCA–itaconate axis is an important mechanistic basis for GC efficacy. Direct evidence in human RA synovial macrophages, however, still awaits validation through longitudinal studies.

Methotrexate (MTX) acts beyond its classical mechanism of adenosine accumulation [147,148,149]. By blocking one-carbon metabolism, it can also remodel the myeloid transcriptome, induce a state of LPS tolerance and downregulate the TLR4 co-receptor sCD14 [150]. In RA patients, serum sCD14 levels in MTX responders decline significantly over the course of treatment. This raises the possibility that baseline sCD14 may help predict the therapeutic response to MTX in early RA, serving as a candidate predictive biomarker [150]. Mechanistically, adenosine accumulation activates the A2AR–cAMP–AMPK axis, which further suppresses pro-inflammatory glycolysis and promotes the restoration of mitochondrial oxidative phosphorylation. Acting together with the blockade of one-carbon metabolism, this constitutes a multi-tiered regulation of the macrophage metabolic programme [149,151].

In summary, glucocorticoids and methotrexate are clinically validated, marketed antirheumatic drugs. They regulate macrophage metabolic reprogramming through the TCA–itaconate axis and the glucose metabolism network, respectively. These findings provide conceptual support for developing next-generation RA interventions that target macrophage metabolism. Even so, the path from animal models and clinical correlative evidence to genuine metabolism-targeted therapy remains long. It will still require metabolomic and functional validation at the level of synovial biopsy.

### 6.2. Emerging Metabolic Inhibitors and Pathway-Directed Interventions

Although b/tsDMARDs—including TNF-α antagonists, IL-6 receptor antagonists, B-cell-depleting agents, and JAK inhibitors—have markedly improved outcomes in RA [152,153,154], approximately 30–40% of patients fail to achieve an adequate initial response or subsequently lose treatment response [155,156]. Long-term therapy may also be constrained by class-specific safety concerns, including opportunistic infections [157,158], cardiovascular events [159,160], and high relapse rates following treatment withdrawal [161,162]. Most available therapies suppress cytokine networks or intracellular immune signalling rather than directly modifying the metabolic programmes associated with persistent pathogenic macrophage states. This distinction has raised interest in macrophage metabolism as a complementary therapeutic layer rather than a substitute for established RA treatment. Although current evidence is insufficient to establish macrophage metabolic reprogramming as an independent upstream driver of RA, several metabolic checkpoints have been implicated in disease progression, providing a preclinical rationale for the development of pathway-directed inhibitors.

On this basis, recent studies have identified potentially targetable metabolic enzymes at multiple levels, including glycolysis, the hexosamine biosynthetic pathway, and arginine metabolism-related post-translational modifications. The therapeutic potential of corresponding small-molecule inhibitors has been evaluated preliminarily in animal models and in vitro cellular systems. Representative metabolic inhibitors, experimental models and dosing regimens are summarised in Table 2.

Although these targets are supported by mechanistically compelling preclinical evidence, none of the compounds listed in Table 2 has entered a registered clinical trial for RA, and each faces distinct translational barriers. SR9243 and OSMI-1 remain research tool compounds. The development of SR9243 is constrained by concerns regarding lipid dysregulation and hepatotoxicity associated with LXR modulation, whereas OGT, the target of OSMI-1, is essential for cell survival. Among these agents, 2-DG has the most extensive clinical safety data; however, dose-limiting hypoglycaemia and electrocardiographic QT-interval prolongation make it poorly suited to the prolonged treatment required for a chronic disease such as RA. BB-Cl-amidine, a covalent pan-PAD inhibitor, has limited selectivity and may compromise neutrophil-mediated host defence. Although bexarotene is already approved for clinical use, severe hypertriglyceridaemia, central hypothyroidism, and teratogenicity limit its suitability for long-term treatment in RA. Collectively, the targets of these inhibitors—including GLUT1, HK2, LXR, OGT, and PAD family members—are widely expressed across tissues and perform essential physiological functions. Because the corresponding agents lack cellular or tissue selectivity, systemic administration is unlikely to achieve an acceptable balance between efficacy and safety. Future strategies integrating intra-articular delivery, cell-selective targeting, and targeting approaches informed by pathogenic macrophage subsets identified through single-cell omics, together with metabolic biomarkers for patient stratification, may allow the clinical feasibility of these targets to be reassessed within a narrower and more controllable therapeutic window.

### 6.3. Natural Products as Immunometabolic Modulators

Although widely used DMARDs and biologic agents are effective in controlling inflammation and delaying joint destruction, substantial unmet clinical needs remain. Approximately 40% of patients fail to respond to monotherapy, and 5–20% are refractory to all currently available drugs [156,165,166]. Alternative therapeutic agents with novel molecular targets are therefore urgently needed. Natural products may provide an important source of such agents. Their multitarget properties enable them to modulate macrophage glycolysis, lipid metabolism, and metabolism-associated epigenetic modifications. However, the supporting evidence is derived predominantly from in vitro experiments and animal models. Representative natural products, experimental models, dosing regimens and proposed metabolic mechanisms are summarised in Table 3.

Some natural products primarily affect glycolysis and metabolism-associated epigenetic regulation. In AA and CIA models, as well as in cultured macrophages, berberine activates AMPK and inhibits mTORC1–HIF-1α signalling. These effects reduce glucose uptake, lactate production, and pro-inflammatory macrophage programmes [167,168]. Berberine also modulates p300-mediated acetylation of NF-κB p65. This results in the nuclear accumulation of p65 in an acetylated, non-phosphorylated form, thereby impairing its activity as a transcriptional activator. Consequently, the transcription of M1-associated pro-inflammatory cytokines is suppressed [169]. Geniposidic acid has been reported to reduce FLS exosome-induced H3K56 lactylation through an ACLY/HDAC6-related mechanism. This effect suppresses pro-inflammatory gene expression in macrophages and attenuates synovial inflammation [170]. Collectively, these studies suggest that natural products may regulate not only metabolic flux but also metabolite-dependent chromatin processes.

A second group of interventions primarily affects lipid storage and lipid-mediator networks. In preclinical models, Sanmiao Pill promotes PPARγ nuclear translocation and inhibits HILPDA/DGAT1-associated lipid-droplet accumulation. These effects are accompanied by reduced activity of the cPLA_2_, COX-2, and PGE_2_ pathways [171]. In human monocyte-derived macrophages, Tripterygium wilfordii glycosides induce state-dependent remodelling of lipid-mediator profiles. Under pro-inflammatory conditions, they suppress the production of 5-LOX-related lipid mediators. Under alternatively activated conditions, they enhance the generation of 12/15-LOX-related pro-resolving mediators [172]. The combination of Wuwei Ganlu and myricetin inhibits SHBG/SREBP1-associated fatty acid synthesis and lipid accumulation in IL-1Ra-deficient mice and RAW264.7 cells [173]. Nevertheless, these findings are derived mainly from rodent models of arthritis and in vitro cellular systems. Macrophage-specific causal validation, metabolic-flux analysis in human synovial tissue, and prospective clinical studies remain lacking.

**Table 3:** Natural Products Modulating Macrophage Metabolism in Experimental Arthritis.

Metabolic Reprogramming	Intervention	Model System	Dosing Regimen	Mechanisms
Glycolysis	Berberine [167]	AA rats, PMs	In vivo: 40, 80, or 160 mg/kg/day, oral gavage, for 14 days In vitro: 10 μM for 24 h	Alleviates joint inflammation and balances macrophage polarization by activating AMPK to suppress HIF-1α-mediated energy metabolism.
	Berberine [168]	CIA mice, BMDMs, RAW 264.7	In vivo: 100 or 200 mg/kg/day, oral gavage, for 21 days In vitro: 20 μM for 24 h	Restores the M1/M2 macrophage balance by activating AMPK, thereby inhibiting the mTORC1/HIF-1α axis and suppressing glycolytic reprogramming.
	Geniposidic acid [170]	AIA rats, RAW264.7 cells	In vivo: 50 or 100 mg/kg/day, oral gavage, for 50 days In vitro: 16 or 32 μM; exposure time NR	Ameliorates RA by targeting the ACLY/HDAC6 axis to suppress histone H3K56 lactylation, thereby inhibiting M1-type pro-inflammatory gene transcription.
Lipid metabolism	Wuwei Ganlu and Myricetin [173]	IL-1Ra^−^/^−^ mice, RAW 264.7	In vivo: myricetin, 50 or 100 mg/kg/day, orally, for 28 days In vitro: Wuwei Ganlu, 10 or 100 μg/mL for 24 h; myricetin, 1–500 ng/mL for 24 h	Ameliorates RA and inhibits M1 macrophage polarization by targeting the SHBG/SREBP1 axis to reprogram fatty acid metabolism and restore lipid mediator balance.
	Sanmiao pill [171]	CIA rats, RAW 264.7	In vivo: 2.65, 5.29, or 10.58 g/kg/day, oral gavage, for 14 days In vitro: 5%, 10%, or 15% drug-containing serum; exposure time NR	Alleviates synovitis by promoting PPARγ nuclear translocation, which restricts intracellular lipid droplet accumulation and PGE2 synthesis to suppress M1 polarization.
Amino acid metabolism	β-Sitosterol [174]	CIA mice, BMDMs	In vivo: 20 or 50 mg/kg, i.p., every 2 days from day 0 to day 31 In vitro: 5, 25, or 50 μM for 24–48 h	Promotes M2 polarization by shifting arginine metabolism from iNOS-mediated NO production to Arg-1-mediated polyamine synthesis via an IL-10 dependent manner.
Amino acid/Lipid metabolism	Jinwu Jiangu capsule [175]	CIA rats, THP-1	In vivo: 0.16, 0.49, or 1.47 g/kg/day, oral gavage, for 28 days In vitro: 5%, 15%, or 20% drug-containing serum for 6 h	Restricts M1 macrophage polarization and synovial inflammation by targeting the SLC7A11/GSH/GPX4 pathway to mitigate ROS accumulation and ferroptosis-related lipid metabolic disorders.

### 6.4. Precision Delivery and Nanotherapeutics

Although metabolic inhibitors and natural products have shown anti-inflammatory activity in experimental arthritis, their further development is limited by several factors. These include the widespread tissue expression of their molecular targets, poor drug solubility and bioavailability, and pharmacokinetic incompatibility between agents used in combination. Nanodelivery systems may help address some of these limitations by exploiting molecular markers enriched in the RA synovium, such as CD44 and FRβ, as well as pathological microenvironmental features, including low pH, elevated MMP activity, and increased ROS levels. Such systems can promote lesion-specific accumulation, enhance local drug exposure, and enable the co-delivery of agents with distinct physicochemical properties. Representative nanodelivery platforms, experimental models, dosing regimens and proposed metabolic mechanisms are summarised in Table 4.

Most studies to date have focused on inflammatory macrophages characterized by high glycolytic activity. Carrier-free nanoparticles formed through the self-assembly of curcumin and 2-DG improve curcumin delivery while simultaneously inhibiting GLUT1/HK2-associated glycolysis. In AIA models and RAW264.7 cells, these effects are accompanied by reduced HIF-1α/NF-κB signalling and attenuation of pro-inflammatory macrophage phenotypes [176]. Roburic acid nanoparticles similarly restrict glucose uptake by targeting the ERK–HIF-1α–GLUT1 axis and improve inflammation-associated metabolic abnormalities in AIA rats [177]. A self-nanoemulsifying formulation of tangeretin has also been reported to suppress LDHA-associated lactate production and modulate both glycolysis and the pentose phosphate pathway [178]. Collectively, these studies indicate that nanoformulations can improve the exposure and delivery efficiency of candidate compounds. However, they do not yet establish that these platforms can selectively or durably reprogramme macrophage metabolism in the human RA synovium.

A second group of strategies exploits features of the inflammatory microenvironment to achieve on-demand drug release and combined intervention. HA–TGMS polymersomes co-deliver 2-DG and dexamethasone through pH- and MMP-responsive mechanisms. HA–CD44 interactions further enhance uptake by inflamed tissues and multiple activated cell populations [179]. ROS-responsive artesunate–dexamethasone micelles integrate the modulation of oxidative stress with inhibition of HIF-1α/NF-κB signalling within a single delivery platform [180]. Together, these studies illustrate the potential design advantages of stimulus-responsive co-delivery systems for coordinating drug-release kinetics, lesion localization, and pharmacological synergy.

Overall, nanotechnology is more appropriately viewed as a delivery platform that may improve the local availability and therapeutic window of immunometabolic interventions than as an independently validated therapeutic paradigm. Current evidence is derived primarily from cell lines and rodent models of arthritis. Clinical translation will therefore require careful evaluation of long-term safety, carrier stability, immunogenicity, and other formulation-related challenges. Moreover, most existing studies continue to target broadly defined M1-like macrophages. Future studies could integrate single-cell and spatial multi-omics analyses of human synovial tissue to identify pathogenic macrophage states marked by stable and accessible surface molecules. These findings could, in turn, guide the development of subset-selective delivery strategies.

**Table 4:** Nanodelivery Strategies Modulating Macrophage Metabolism in Experimental Arthritis.

Metabolic Reprogramming	Nanoplatform	Model System	Dosing Regimen	Mechanisms
Glycolysis	RBA-NPs [177]	AIA rats, RAW 264.7, THP-1	In vivo: 5 mg/kg RBA equivalent, i.v., on days 17, 20, 23, and 26 after arthritis induction. In vitro: 20 μM for 24 h; 40 μM was additionally used in dose–response metabolic assays.	Ameliorate RA by inducing macrophage M1 to M2 polarization through the inhibition of ERK/HIF-1α/GLUT1-mediated glycolysis.
	SNEDDS-TG [178]	AIA rats, RAW 264.7	In vivo: 100 mg/kg/day, oral administration, for 28 days. In vitro: 50, 100, or 200 μM for 6 h.	Restricts M1 macrophage polarization by directly antagonizing LDHA activity and downregulating the expression of HIF-1α, G6PD, and PDH in the inflammatory microenvironment.
	DEX/HTA micelles [180]	AIA rats, RAW 264.7	In vivo: 2.5 mg/kg DEX equivalent, i.v., every 3 days for five doses beginning on day 14; ART:DEX ratio, 1:1. In vitro: ART:DEX ratio, 1:1; concentrations NR; exposure time varied from 8 to 72 h according to the assay.	Targeting the HIF-1α/NF-κB signaling cascade, DEX/HTA micelles synergistically regulate ROS scavenging and drive macrophage repolarization.
	2-DG/Cur nanoparticles (2-DCNP) [176]	AIA rats, RAW264.7	In vivo: 120 mg/kg 2-DG equivalent, i.v., every 2 days for 2 weeks. In vitro: concentration NR; 24 h.	Restricts glucose metabolism via GLUT1 and HK2 inhibition, synergistically downregulating the HIF-1α/NF-κB cascade to drive M2 macrophage polarization.
	ADSCs-EXO-ICA [181]	CIA rats, RAW264.7	In vivo: 200 or 400 μg formulation per rat, tail-vein i.v., every other day for eight injections. In vitro: 80 μg/mL for 48 h.	ADSCs-EXO-ICA promotes an M1-to-M2 phenotypic transition by reducing glycolysis through the inhibition of the ERK/HIF-1α/GLUT1 pathway.
	NM@NP-Bex [182]	CIA mice, BMDMs	In vivo: 1 mg/kg bexarotene equivalent, i.v., every 2 days from day 21 to day 48. In vitro: 400 nM bexarotene equivalent during M1/M2 polarization for 48 h.	NM@NP-Bex reverses M1 macrophage glycolytic reprogramming and corrects the M1/M2 imbalance by targeting Pim2 kinase to block the phosphorylation of PGK1, PDHA1, and PFKFB2.
	HTDD polymersomes [179]	AIA rats, RAW264.7	In vivo: Dex 0.75 mg/kg plus 2-DG 8 mg/kg, i.v., on days 10, 12, and 14 after arthritis induction. In vitro: Dex 15 μg/mL plus 2-DG 200 μg/mL for 24 h.	2-DG blocks glycolysis to reduce Dex efflux, while Dex scavenges ROS and suppresses inflammation, synergistically reversing M1 polarization and restoring immune homeostasis.
	DM/SHK [183]	CIA mice, RAW264.7	In vivo: dose NR; i.v., every other day from day 28 to day 44, with sacrifice on day 45. In vitro: concentration NR; 24 h.	Triggers efferocytosis and synergistically activates the AMPK/UCP2 axis to promote OXPHOS, which reprograms M1 macrophages into the pro-resolving M2 phenotype.

## 7. Conclusions and Discussion

Macrophages are no longer regarded as passive responders to inflammation in the rheumatoid synovium. Rather, they are increasingly recognized as dynamic regulators that integrate inflammatory signals, metabolic stress, and tissue-damage programmes, thereby contributing to the persistence of synovial inflammation in RA. A major conceptual advance in this field is the recognition that macrophage metabolic reprogramming is not merely a secondary consequence of inflammation. Instead, it may represent a critical mechanism through which transient inflammatory stimuli are converted into durable pathogenic states. Inflammatory and metabolic pressures within the synovium induce metabolic adaptation in macrophages, and the resulting programmes may, in turn, stabilize disease-associated states by reshaping inflammatory transcription, metabolite-mediated signalling, and intercellular communication. The principal pathological consequence may therefore be the prolonged persistence of pathogenic macrophage states, rendering synovial inflammation less amenable to spontaneous resolution.

A second key advance is the recognition that metabolism does not simply provide permissive conditions for macrophage activation but can actively instruct cellular function. Metabolic pathways determine the availability of ATP and biosynthetic precursors while also shaping chromatin organization, transcriptional programmes, cytokine secretion profiles, and communication with neighbouring cells. These effects are mediated by metabolic intermediates, redox balance, and epigenetic modifications. Macrophage metabolic reprogramming can therefore be viewed as an important mechanistic bridge linking the pathological synovial microenvironment to the acquisition and maintenance of pathogenic effector functions.

Several major challenges nevertheless remain. First, macrophage populations in the RA synovium are highly heterogeneous, and the conventional M1/M2 dichotomy cannot adequately capture the complex combinations of metabolic and functional programmes observed in human disease. Human synovial macrophage states are shaped by cellular origin, spatial niche, and disease stage, and apparently divergent functions may coexist within the same population. For example, SPP1^+^ macrophages associated with active RA exhibit inflammatory and tissue-remodelling features while also showing enrichment of HSD11B1, which encodes an enzyme involved in local cortisol regeneration. Although the functional significance of this observation remains unresolved, it indicates that inflammation-associated macrophages are not defined solely by a unidirectional pro-inflammatory programme. They may simultaneously engage adaptive or compensatory regulatory mechanisms.

Second, many studies of macrophage metabolism continue to rely on in vitro polarization systems or population-level analyses. These approaches cannot fully reproduce the spatial organization, temporal evolution, and multicellular interactions of the native synovial microenvironment. Third, the functional significance of metabolic alterations is likely to vary across disease stages, tissue compartments, and macrophage subsets. Depending on the context, such alterations may act as initiating drivers, amplifiers of persistent inflammation, or adaptive responses to local stress. This complexity also creates substantial translational challenges. Broad inhibition of metabolic pathways may attenuate inflammation, but it may simultaneously compromise tissue repair, host defence, and systemic immune homeostasis.

Overall, current evidence supports an emerging model of RA pathogenesis in which macrophage metabolism is not an ancillary feature of the inflammatory milieu but an important determinant of inflammatory chronicity, stromal activation, and progressive bone damage. This perspective deepens our understanding of RA pathogenesis and positions macrophage immunometabolism as a potential entry point for therapeutic innovation. It also raises a central hypothesis: if pathogenic macrophage states depend on local inflammatory and metabolic programmes for their maintenance, then directly modulating macrophage metabolism—or reshaping the cellular niche by disrupting pathological interactions with other synovial cell populations—may alter macrophage state transitions and facilitate the restoration of synovial homeostasis. This hypothesis provides a conceptual basis for considering longitudinal synovial studies, niche-directed interventions, and the requirements for clinical translation.

## 8. Future Perspectives

Future research should move beyond simply expanding the catalogue of metabolic abnormalities in RA macrophages. Greater emphasis should instead be placed on the mechanisms governing transitions between macrophage states and on whether this plasticity can be harnessed to restore synovial homeostasis. Current evidence is insufficient to define macrophage metabolic reprogramming as an independent driver of RA initiation. Nevertheless, it supports a role for metabolic reprogramming in stabilizing inflammatory cell states, sustaining pathological cellular interactions, and limiting the resolution of inflammation. A central question therefore requires direct investigation: can pathogenic macrophage programmes be attenuated, and the resident macrophage-supported synovial barrier and pro-resolving network restored, by directly modulating macrophage metabolism or by altering the local inflammatory and metabolic environment that continually shapes macrophage states?

Addressing this question will require longitudinal studies in human disease. Paired synovial biopsies collected before and after treatment could determine whether inflammatory, tissue-remodelling, and interferon-responsive macrophage states undergo transition and whether homeostasis-associated programmes marked by FOLR2^+^, MerTK^+^, and LYVE1^+^ cells are restored. In this context, the principal value of single-cell and spatial multi-omics is not merely to identify additional cell subsets. Rather, these approaches can link macrophage states to their anatomical location, metabolic activity, and interactions with neighbouring cells. Their integration with metabolite imaging, metabolic-flux analysis, and functional perturbation of human synovial tissue may help distinguish transient inflammatory suppression from durable remodelling of the synovial cellular niche.

Future interventions should also shift away from prolonged systemic inhibition of fundamental metabolic pathways towards strategies with greater tissue, cell-state, and disease-stage selectivity. Intra-articular delivery, time-limited metabolic interventions, and agents directed against specific macrophage states or the signals that sustain them may widen the therapeutic window. Such approaches may also limit adverse effects on host defence, tissue repair, and systemic metabolic homeostasis. Macrophage metabolism could additionally be modified indirectly by targeting other synovial cell populations involved in pathogenic interactions with macrophages. Chimeric antigen receptor T cell (CAR-T) therapies targeting CD19 or CD19/CD20 have shown early clinical signals in a small number of patients with refractory RA [184,185,186,187]. Observations that B-cell reconstitution does not necessarily coincide with disease relapse suggest that profound B-cell depletion may reshape the autoreactive B-cell compartment [187,188]. However, no direct evidence currently shows that this effect alters synovial macrophage metabolic programmes by reducing autoantibodies, immune complexes, or related inflammatory stimuli. The proposed relationship between CAR-T-cell therapy and macrophage immunometabolism should therefore be regarded as a testable hypothesis rather than an established therapeutic mechanism.

Candidate biomarkers may provide a clinically accessible, although indirect, window into the immunometabolic state of the synovium. In the near term, these biomarkers are more likely to assist disease stratification, risk assessment, and pharmacodynamic monitoring than to determine treatment selection independently. Circulating soluble PKM2 may serve as a candidate marker of glycolytically active inflammation and structural damage risk [189]. Synovial programmes associated with FOLR2, MerTK, and LYVE1 may reflect the integrity of resident macrophage networks and the extent of local homeostatic restoration. Monocyte- or macrophage-associated interferon signatures may indicate persistent myeloid inflammation and variation in treatment response [48,190]. No single marker, however, is likely to capture the spatial and cellular heterogeneity of the RA synovium. A more feasible strategy may therefore be to integrate circulating metabolic biomarkers, synovial molecular pathology, and subclinical inflammation detected by ultrasonography or magnetic resonance imaging. Such a multimodal framework could help distinguish persistent pathological activity from genuine restoration of tissue homeostasis.

On this basis, macrophage-directed metabolic interventions may be particularly relevant for investigation in patients with persistent myeloid-dominant synovitis, a high risk of structural damage, or an inadequate response to guideline-recommended DMARD therapy. Patients with residual subclinical synovitis despite clinical remission may also constitute an informative population in which to investigate the relationship between macrophage homeostatic programs and disease flare. At present, however, these features should be regarded only as enrichment criteria for prospective studies, not as established criteria for treatment selection. Within current treatment paradigms, macrophage metabolic interventions should initially be evaluated as adjunctive or biomarker-guided strategies alongside established RA therapies, rather than as stand-alone replacements. In addition to assessing disease activity, sustained remission, and structural progression, future trials should evaluate treatment-associated changes in macrophage states, spatial cellular interactions, and metabolism-related biomarkers. This would help determine whether clinical benefit is accompanied by durable remodeling of the synovial immunometabolic environment.

Overall, the most consequential advances may arise not from identifying additional isolated metabolic targets, but from defining the key metabolic and intercellular interaction nodes that sustain pathogenic macrophage states. Equally important will be determining how these nodes can be modulated with tissue and disease-stage selectivity. Integrating longitudinal synovial studies, functional metabolic analyses, and clinical phenotyping will be essential for defining the role of macrophage metabolic regulation in RA treatment and identifying the clinical settings in which it is most likely to provide meaningful benefit.

## Figures and Tables

**Table 1:** Phenotypic classification and functional characteristics of macrophages in RA.

Macrophage Subsets	Inducing Stimuli	Surface Marker	Cytokine Profiles	Functions
Pro-inflammatory phenotypes				
M1 [23,24,25]	LPS, TH1 cytokines (IFN-γ, TNF-α)	CD80, CD86, iNOS, TLR-2, MHC-II	iNOS, IL-1β, IL-12, TNF-α	Pro-inflammatory, antigen presentation, activation of T cells and FLSs, amplification of active RA synovitis and bone damage.
AtoMs [26,27]	TNF, RANKL, GM-CSF, IL-1β	CX3CR1^+^, CD14^+^, CD64^+^, CD80^+^, CD86^+^, ±CD11c, FoxM1^+^	TNF, IL-1β, IL-6	Osteoclast precursors, promote osteoclast differentiation, contribute to bone erosion, local inflammation and T-cell activation in RA.
Anti-inflammatory phenotypes				
M2a [28,29]	IL-4, IL-13	CD206, IL-1R, Arg-1	IL-10, TGF-β, CCL17, CCL18, Arg-1, Fibronectin	Tissue repair, extracellular matrix remodelling, fibrosis and resolution-associated responses, produces anti-inflammatory mediators such as IL-10 and TGF-β.
M2b [30,31]	TLR ligands, immune complexes, IL-1β	CD86, IL-6R, IL-10R	IL-10, IL-6, IL-1β	Immunoregulatory but mixed phenotype; modulates inflammatory responses and T helper cell balance in RA.
M2c [32,33]	IL-10, TGF-β, glucocorticoids	CD163, CD206, MerTK, TLR1, TLR8	IL-10, TGF-β, CCL16, CCL18, CXCL13	Tissue repair, resolution of inflammation, cellular debris clearance; generally present in the remission phase of RA.
Other/Intermediate phenotypes				
Hybrid M1-M2 [34,35]	Mixed cytokines (e.g., IFN-γ + IL-4 or IL-10), chronic inflammation	Co-expression of CD80, CD163, CD204, TLR4	IL-10, IL-6, TGF-β	Context-dependent inflammatory or tissue-remodelling functions; reflects continuum-like macrophage activation in RA synovium.

**Table 2:** Preclinical evidence for key metabolic reprogramming target inhibitors in macrophages.

Metabolic Target	Inhibitors	Model System	Dosing Regimen	Mechanisms
Liver X receptor	SR9243 [84]	AIA rats, RAW264.7	In vivo: 15 or 30 mg/kg/day, i.p., for 27 days In vitro: 5–20 μM for 6 h	Inhibits M1 macrophage polarization and glycolysis via the AMPK/mTOR/HIF-1α pathway.
HK2	2-DG [163]	AA rats, RAW264.7	In vivo: 50–200 mg/kg/day, oral gavage, for 14 days In vitro: 0.4 mM for 24 h following LPS stimulation	2-DG inhibits glycolysis, activates AMPK, and suppresses NF-κB, reprogramming macrophages from M1 to M2 phenotype and ameliorating arthritis in an AMPK-dependent manner.
PAD2	BB-Cl-amidine [164]	THP-1	In vitro: 100 nM; 30 min pretreatment followed by 48 h co-incubation	BB-Cl-amidine inhibition of PAD2 downregulates NF-κB pathway proteins and reduces TXNIP/Caspase-1 expression, skewing macrophages from the M1 to M2 phenotype.
Ogt	OSMI-1 [94]	LPS-induced calvarial bone-loss model; murine BMM	In vivo: 10 mg/kg/day, s.c. over the calvaria, for 6 days In vitro: 1–25 μM for 96 h in the presence of M-CSF and RANKL	OSMI-1 inhibition of Ogt reduces O-GlcNAcylation, blocking nuclear translocation of both transcription factors and suppressing osteoclastogenesis in vitro and in vivo.

## Data Availability

No new data were created or analyzed in this study.

## Abbreviations

11β-HSD	11β-hydroxysteroid dehydrogenase
2-DCNP	Carrier-free nanoparticles
2-DG	2-deoxy-d-glucose
4-OI	4-octyl itaconate
5-LOX	5-lipoxygenase
A2AR	Adenosine A2A receptor
AA	Arachidonic acid
aaRSs	Aminoacyl-tRNA synthetases
ACOD1	Aconitate decarboxylase 1
ACPAs	Anti-citrullinated protein antibodies
AIA	Adjuvant-induced arthritis
α-KG	α-ketoglutarate
AP-1	Activator protein 1
Arg-1	Arginase-1
AtoMs	Arthritis-associated osteoclastogenic macrophages
BLT1	Leukotriene B4 receptor 1
BMDMs	Bone marrow-derived macrophages
b/tsDMARDs	Biologic and targeted synthetic disease-modifying antirheumatic drugs
C5a	Complement component 5a
C5aR	Complement component 5a receptor
cAMP	Cyclic adenosine monophosphate
CAR-T	Chimeric antigen receptor T cell
CCL2	C-C motif chemokine ligand 2
CCR1	C-C chemokine receptor type 1
CD48	Cluster of differentiation 48
CIA	Collagen-induced arthritis
COX	Cyclooxygenases
COX-2	Cyclooxygenase 2
COX4	Cytochrome c oxidase subunit 4
cPLA2	Cytosolic phospholipase A2
CREB	cAMP response element-binding protein
csDMARD	Conventional synthetic DMARD
CXCR2	C-X-C chemokine receptor type 2
CYP450	Cytochrome P450
D2T RA	Difficult-to-treat rheumatoid arthritis
DAMPs	Damage-associated molecular patterns
Dex	Dexamethasone
DEX/HTA	Artesunate-dexamethasone co-loaded micelles
DMARDs	Disease-modifying anti-rheumatic drugs
ECM	Extracellular matrix
ELMO1	Engulfment and cell motility 1
EP4	Prostaglandin E2 receptor 4
ETC	Electron transport chain
FAMIN	Fatty acid metabolism–immunity nexus
FAO	Fatty acid oxidation
FcγRs	Fcγ receptors
FLS	Fibroblast-like synoviocytes
Flt-1	FMS-like tyrosine kinase 1
GCs	Glucocorticoids
GDH	Glutamate dehydrogenase
GLUT1	Glucose transporter 1
GR	Glucocorticoid receptor
GRK2	G protein-coupled receptor kinase 2
H3K18la	Histone H3 lysine 18 lactylation
H3K56la	Lactylation of histone H3 at lysine 56
HA-TGMS	Hyaluronic acid–triglycerin monostearate
HBP	Hexosamine biosynthesis pathway
HETEs	Hydroxyeicosatetraenoic acids
HIF-1α	Hypoxia-inducible factor 1α
HK2	Hexokinase 2
HMOX1	Heme oxygenase 1
HTDD	HA-TGMS polymersomes
IFN	Interferon
IκBζ	NF-κB inhibitor ζ
IL-1β	Interleukin-1β
IL-6	Interleukin-6
iNOS	Inducible nitric oxide synthase
IRG1	Immune-responsive gene 1
JIA	Juvenile idiopathic arthritis
KDM6A	Lysine-specific demethylase 6A
KEAP1	Kelch-like ECH-associated protein 1
LACC1	Laccase domain-containing 1
LDH	Lactate dehydrogenase
lncRNA	Long non-coding RNA
LOX	Lipoxygenases
LPS	Lipopolysaccharide
LTB4	Leukotriene B4
MerTK	MER proto-oncogene tyrosine kinase
METs	Macrophage extracellular traps
MMPs	Matrix metalloproteinases
MTX	Methotrexate
NFATc1	Nuclear factor of activated T cells cytoplasmic 1
NF-κB p65	Nuclear factor κB p65 subunit
NLRP3	NOD-like receptor family pyrin domain-containing 3
NM@NP-Bex	Neutrophil membrane-biomimetic nanodelivery system
NO	Nitric oxide
NQO1	NAD(P)H quinone dehydrogenase 1
Nrf2	Nuclear factor erythroid 2-related factor 2
OCM	One-carbon metabolism
ODC1	Ornithine decarboxylase 1
OGT	O-GlcNAc transferase
OSMI-1	Small-molecule OGT inhibitor 1
OXPHOS	Oxidative phosphorylation
PADs	Peptidylarginine deiminases
PDH	Pyruvate dehydrogenase
PFKFB3	6-phosphofructo-2-kinase/fructose-2,6-biphosphatase 3
PGE2	Prostaglandin E2
PHD	Prolyl hydroxylase domain
PKA	Protein kinase A
PKM2	Pyruvate kinase M2
PPARγ	Peroxisome proliferator-activated receptor γ
PPP	Pentose phosphate pathway
RA	Rheumatoid arthritis
ROS	Reactive oxygen species
S100A12	S100 calcium-binding protein A12
scRNA-seq	Single-cell RNA sequencing
SDH	Succinate dehydrogenase
SHBG	Sex hormone-binding globulin
SPP1	Secreted phosphoprotein 1
SREBP1	Sterol regulatory element-binding protein 1
STAT	Signal transducer and activator of transcription
STMs	Synovial tissue macrophages
SUCNR1	Succinate receptor 1
TCA	Tricarboxylic acid
TGF-β	Transforming growth factor-β
TLR2/4	Toll-like receptors 2/4
TNF-α	Tumor necrosis factor-α
TRE	TPA-responsive element
TREM2	Triggering receptor expressed on myeloid cells 2
TXA2	Thromboxane A2
UDP-GlcNAc	Uridine diphosphate N-acetylglucosamine
ΔΨm	Mitochondrial membrane potential
